# Maintaining Adherence Programme: evaluation of an innovative service model

**DOI:** 10.1192/pb.bp.114.048496

**Published:** 2016-02

**Authors:** Llewellyn Lewis, Christine O'Keeffe, Ian Smyth, Judi Mallalieu, Laura Baldock, Sam Oliver

**Affiliations:** 1 South Essex Partnership University NHS Foundation Trust, Southend, UK; 2 Janssen Healthcare Innovation, High Wycombe, UK; 3 pH Associates, Marlow, UK

## Abstract

**Aims and method** The Maintaining Adherence Programme (MAP) is a new model of care for patients with schizophrenia, schizoaffective disorder and bipolar affective disorder which aims to encourage adherence and prevent relapse. This evaluation, conducted by retrospective and prospective data collection (including patient questionnaires and staff interviews), aimed to describe MAP's impact on healthcare resource use, clinical measures and patient and staff satisfaction, following its implementation in a university National Health Service (NHS) foundation trust in England. We included 143 consenting patients who entered MAP before 31 March 2012.

**Results** In-patient bed days and non-MAP NHS costs reduced significantly in the 18 months post-MAP entry. At 15–18 months post-MAP, Medication Adherence Rating Scale scores had improved significantly from baseline and there was a shift towards less severe clinician-rated disease categories. Based on patient surveys, 96% would recommend MAP to friends, and staff were also overwhelmingly positive about the service.

**Clinical implications** MAP was associated with reduced cost of treatment, improvements in clinical outcomes and very high patient and staff satisfaction.

Non-adherence to prescribed medication is common in conditions such as schizophrenia and bipolar affective disorder^1,2^ and has important implications for patients, their families, healthcare providers and society. Non-adherence increases the risk of relapse, rehospitalisation and suicide^3,4^ and is linked to impaired long-term functional outcomes.^5^ The impact on direct healthcare costs, already significantly higher in this patient group than in other psychiatric populations,^6^ is considerable.^7,8^ Interventions which target non-adherence to treatment overall therefore have the potential to make significant impact on both healthcare resource use and patient outcomes.

In 2011, South Essex Partnership University NHS Foundation Trust (SEPT) implemented a new model of care (the Maintaining Adherence Programme – MAP) for patients with schizophrenia, bipolar affective disorder and schizoaffective disorder. Its aim is to encourage treatment adherence, prevent relapse and ultimately reduce rehospitalisation costs. This was achieved through a joint working agreement between the trust and Janssen Healthcare Innovation. The programme centres around the provision of structured, in-depth psychoeducation, an evidence-based approach which has been shown to reduce relapse and encourage medication adherence,^9^ with a number of other targeted interventions and support activities to increase engagement. MAP is based on the Munich Adherence Programme, an approach pioneered in Germany. An evaluation of the Munich Adherence Programme Pilot demonstrated a 73% reduction in in-patient bed day use and estimated savings of €5000 per patient over 12 months.^10^

This local service evaluation aimed to describe the impact of MAP when implemented within SEPT to inform local decision-making about ongoing service provision. The primary objective was to evaluate the impact on healthcare resource use. Secondary objectives were to evaluate changes in clinical measures and to assess patient and staff satisfaction.

## Method

Patients eligible for MAP were those with schizophrenia, schizoaffective disorder or bipolar affective disorder, with one or more in-patient admissions (or equivalent stay in an assessment unit or episode with crisis resolution home treatment (CRHT)) in the previous 2 years. Patients with a primary diagnosis of intellectual disability, substance misuse or personality disorder were excluded.

MAP comprised:

- assessment and regular screening of non-adherence by a dedicated adherence team
- structured in-depth psychoeducation (condition-specific) for patients and carers
- well-being activities
- shared decision-making
- telephone and text reminder services
- direct consultant access instead of out-patient clinics
- supportive ‘recovery lounge’ environment.

The ‘core’ programme consisted of 10–11 (depending on diagnosis) condition-specific psychoeducation sessions. Beyond this, MAP structure was highly individualised, following a baseline assessment of the risks associated with non-adherence to treatment and ongoing review of progress. Minor adaptations were made from the Munich Adherence Programme: unlike it, the MAP did not include financial incentives for patients or referring clinicians and depot injection clinics were not introduced specifically for the programme (these were already well established in SEPT).

### Evaluation

The local service evaluation was undertaken between June 2011 and September 2013; it was not part of MAP itself but an independent evaluation of its impact. Patients who entered MAP between 1 June 2011 and 31 March 2012 and provided written informed consent to allow the collection and release of their anonymised data were included (consent rate was 99%, 143/145). Trust research governance approval was obtained.

Based on the results of the Munich Adherence Programme evaluation,^10^ a sample size of 150 was chosen to show the expected mean reduction in bed days of 150 days (95% CI 141–159). This was felt to be both achievable and sufficiently accurate.

Data were collected for each patient both retrospectively (18 months pre-MAP entry date) and prospectively (18 months post-MAP entry date) to allow within-patient comparison of resource use. Eighteen months was chosen to ensure that natural fluctuations in resource use would be observed in both periods.

### Data collection

Baseline demographic characteristics and healthcare resource use for the 18 months pre- and post-MAP entry date were obtained from SEPT's National Health Service (NHS) electronic databases. Details of psychiatric medications prescribed during the programme were collected retrospectively from patients' medical records.

Two measures were administered at baseline and approximately 3-monthly intervals thereafter: the validated, patient-reported Medication Adherence Rating Scale (MARS; scored 0–10, with scores <6 indicating poor adherence)^11^ and the Clinical Global Impressions (CGI) severity,^12^ a clinician-rated measure of mental illness severity. These scores and the frequency and nature of MAP contacts were entered into a specifically designed electronic data collection tool by healthcare professionals delivering MAP.

Patients completed short satisfaction questionnaires approximately 6, 12 and 18 months after MAP entry. Paper questionnaires were provided during routine visits and completed in the clinic.

Staff satisfaction was assessed by conducting one-to-one semi-structured interviews with all staff who had been involved in delivering MAP, when it had been running for approximately 12 months. These were undertaken either face-to-face or by telephone by an independent researcher. Written informed consent was sought to conduct and audio-record the interviews. Interviews were transcribed from the audio recordings and sent to participants for approval before qualitative analysis.

At the end of the evaluation period, pseudonymised data from all sources were sent to the external agency providing evaluation support, for data analysis and reporting.

### Data analysis

The data-set was analysed using Microsoft Excel and Winstat for Excel, according to endpoints pre-specified in the protocol. Descriptive data are presented using the mean (s.d.), median (range) or percentages, as appropriate.

The Student's *t*-matched pairs test or Wilcoxon signed-rank matched pairs test (as appropriate according to data distribution) were applied to assess the significance of the mean within-patient change in each resource use parameter (and overall NHS costs) between the 18 months pre- and the 18 months post-MAP entry; also the change in MARS from baseline at 15–18 months after MAP entry. The Wilcoxon test was used to compare patients' CGI severity categories at baseline and 15–18 months post-MAP entry.

The costs applied to MAP and non-MAP contacts, to assess the impact on overall NHS resource use and estimate the cost of the programme, are shown in Table 1.

**Table 1:** Costs applied to MAP and non-MAP contacts

	Cost per patient (per session/contact unless specified)	
Type of resource	Non-MAP NHS contacts^a^	MAP contacts^b^
In-patient hospitalisation	£327 per day	–

CMHT attendance (duty and non-duty team)	£220	–

Assertive outreach	£172	–

Out-patient appointment	£205.02	–

Assessment unit	£1,419 per episode	–

CRHT	£195.68 per day	–

Resource therapy	£220	–

Initial assessment	–	£175.50

Psychoeducation	–	£26.81

Booster psychoeducation	–	£35.75

Carer psychoeducation	–	£42.90

Follow-up assessment	–	£175.50

Medical review	–	£204.90

Wellness activity	–	£13.00

CMHT, community mental health team; CRHT, crisis resolution home treatment; MAP, Maintaining Adherence Programme; NHS, National Health Service.

a. Based on agreed local reference costs.

b. Based on number and grades of healthcare professionals required to deliver the session and number of attendees.

Analyses are presented for the whole sample, with subanalyses for patients who remained in the programme for the entire 18-month evaluation period (cohort 1) and those who left within 18 months (cohort 2).

Staff interview transcripts were analysed thematically by familiarisation with the texts followed by initial grouping of statements under the broad headings of the interview schedule, within which a number of themes were identified.

## Results

### Evaluation sample

**Fig. 1:**
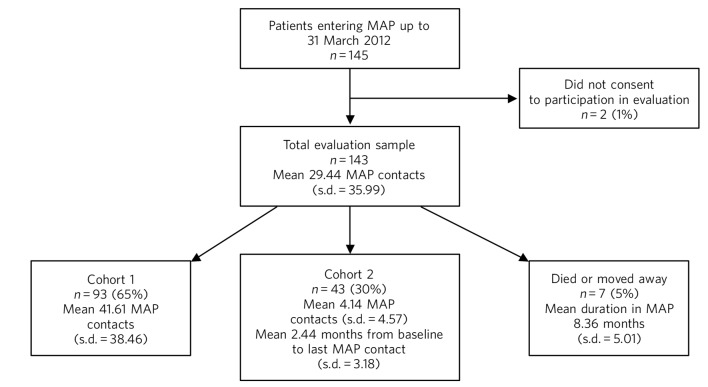
Evaluation sample. MAP, Maintaining Adherence Programme.

In total, 143 patients agreed to participate in the evaluation; patient flow is shown in Fig. 1. Ninety-three patients (65%) either remained in the programme for the entire 18-month evaluation period (*n* = 91) or were discharged within 18 months due to successful treatment (*n* = 2). Forty-three (30%) left the programme within 18 months (mean 4.14 (s.d. = 4.57) MAP contacts per patient; 2.44 (s.d. = 3.18) months from baseline to last contact) and continued to receive conventional care. Seven patients (5%) were lost to follow-up within 18 months owing either to death or moving out of area.

Overall, 55% of patients were male and the mean age at MAP entry was 45.51 years (s.d. = 11.28); 59% of patients had schizophrenia, 27% bipolar affective disorder and 13% schizoaffective disorder. These and other patient characteristics are shown in Table 2.

**Table 2:** Baseline patient characteristics

Baseline patient characteristics	Total evaluation sample (*n* = 143)	Cohort 1 (*n* = 93)	Cohort 2 (*n* = 43)
Male, *n* (%)	78 (55)	45 (48)	29 (67)

Age at programme entry, years: mean (s.d.)	45.51 (11.28)	45.49 (11.03)	45.89 (11.68)

Diagnosis, *n* (%)			
Schizophrenia	84 (59)	52 (56)	27 (63)
Schizoaffective disorder	19 (13)	14 (15)	5 (12)
Bipolar affective disorder	38 (27)	27 (29)	9 (21)
Not recorded^a^	2 (1)	0 (0)	2 (5)

Referral source, *n* (%)			
Adult CMHT	103 (72)	68 (73)	29 (67)
In-patient ward	14 (10)	7 (8)	6 (14)
Assertive outreach	13 (9)	10 (11)	3 (7)
Out-patient	9 (6)	5 (5)	4 (9)
Other	4 (3)	3 (3)	1 (2)

Mental Health Act applications, *n* (%)			
At any time prior to or at programme entry	44 (31)	22 (24)	17 (40)
Current at programme entry	7 (5)	6 (6)	0 (0)

CTOs, *n* (%)			
At any time prior to or at programme entry	9 (6)	4 (4)	5 (12)
Current at programme entry	5 (3)	3 (3)	2 (5)

Hospital in-patient at time of programme entry	18 (13)	12 (13)	5 (12)

CMHT, community mental health team; CTO, community treatment order.

a. Patients discharged from Maintaining Adherence Programme due to change in diagnosis.

### Resource use

**Fig. 2:**
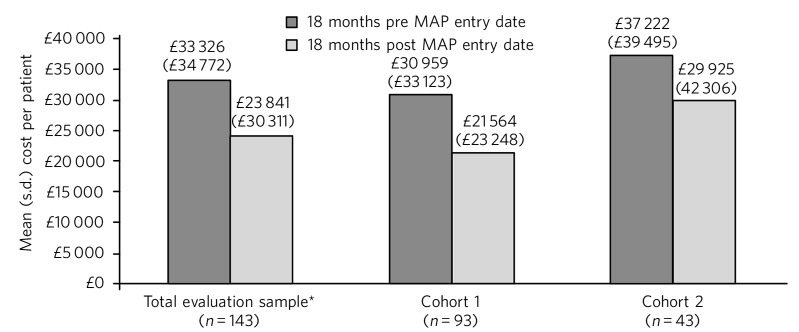
Total cost of non-MAP NHS resource use. *The total evaluation sample includes 7 patients for whom 18 months' follow-up data were not available (because the patient died or moved away). Excluding these patients (*n* = 136), the mean cost of non-MAP NHS resource was £32 939 (s.d. = £35 235) in the 18 months pre-MAP and £24 207 (s.d. = £30 666) in the 18 months post-MAP, a mean reduction of £8732 (s.d. = £34 918) per patient (*P* = 0.002).

In the total evaluation sample, non-MAP NHS costs reduced from a mean of £33 326 (s.d. = £34 772) per patient in the 18 months pre-MAP to £23 841 (s.d. = £30 311) in the 18 months post-MAP; a significant reduction of £9485 (s.d. = £34 785; *P* = 0.001). A significant reduction was also observed in cohort 1 (mean £9396 (s.d. = £34 444), *P* = 0.005), but the mean cost reduction of £7297 (s.d. = £36 293) per patient in cohort 2 was not statistically significant (Fig. 2). The cost of delivering MAP was estimated at £1232, £1708 and £270 (for the total evaluation sample, cohort 1 and cohort 2, respectively), equating to net cost reductions of £8253, £7688 and £7027 per patient.

The number of in-patient bed days per patient reduced significantly in the 18 months post-MAP entry, both in the total evaluation sample (by 24.31 (s.d. = 100.16) days per patient, *P* = 0.002) and in cohort 1 (a reduction of 26.26 (s.d. = 106.64) days per patient, *P* = 0.01). In the total sample there were also significant reductions in assessment unit, assertive outreach, community mental health team (CMHT) duty and out-patient contacts and in cohort 1 in assessment unit and out-patient contacts (Table 3).

**Table 3:** Non-MAP NHS resource in the 18 month period pre and post-MAP entry date

	Total evaluation sample (*n* = 143)			Cohort 1 (*n* = 93)			Cohort 2 (*n* = 43)		
Resource type	Pre-MAP^a^	Post-MAP^b^	Change^c,d^	Pre-MAP^a^	Post-MAP^b^	Change^c,d^	Pre-MAP^a^	Post-MAP^b^	Change^c,d^
In-patient bed days	57.38 (101.84)	33.07 (80.67)	𢈒24.31 (100.16)**	51.01 (100.13)	24.75 (53.85)	𢈒26.26 (106.64)*	69.91 (112.15)	55.21 (121.92)	𢈒14.70 (92.10)

Assessment unit attendances	0.44 (0.88)	0.27 (0.75)	𢈒0.17 (0.80)**	0.34 (0.70)	0.20 (0.50)	𢈒0.14 (0.70)*	0.67 (1.19)	0.44 (1.14)	𢈒0.23 (1.00)

Assertive outreach contacts	8.49 (31.46)	4.91 (16.10)	𢈒3.58 (23.41)*	8.19 (29.23)	5.68 (17.38)	𢈒2.52 (22.45)	10.51 (38.19)	4.00 (14.44)	𢈒6.51 (27.10)

Days on CRHT	10.01 (26.28)	12.93 (38.19)	2.92 (39.79)	10.01 (22.33)	9.75 (26.68)	𢈒0.26 (20.26)	6.23 (14.27)	14.95 (42.57)	8.72 (42.56)

CMHT duty team contacts (urgent)	0.21 (0.78)	0.06 (0.46)	𢈒0.15 (0.92)*	0.22 (0.62)	0.09 (0.56)	𢈒0.13 (0.86)	0.23 (1.09)	0.02 (0.15)	𢈒0.21 (1.10)

CMHT contacts (routine)	28.95 (25.35)	28.45 (23.87)	𢈒0.50 (23.40)	29.08 (27.75)	31.08 (26.02)	2.00 (25.27)	27.00 (20.00)	24.49 (18.34)	𢈒2.51 (16.98)

Resource therapy contacts	15.50 (36.43)	11.10 (31.40)	𢈒4.40 (29.19)*	14.67 (36.30)	13.11 (35.49)	𢈒1.56 (30.80)	17.19 (39.12)	7.74 (23.12)	𢈒9.44 (25.60)*

Out-patient appointments	3.38 (2.42)	2.69 (2.12)	𢈒0.70 (2.35)***	3.67 (2.49)	2.71 (2.19)	𢈒0.96 (2.47)***	2.95 (2.31)	2.60 (1.81)	𢈒0.35 (2.00)

CMHT, community mental health team; CRHT, crisis resolution home treatment; MAP, Maintaining Adherence Programme; NHS, National Health Service.

a. Mean (s.d.) number of each type of contact per patient in the 18 months before the date of MAP entry.

b. Mean (s.d.) number of each type of contact per patient in the 18 months after the date of MAP entry.

c. Mean (s.d.) within-patient change in each type of contact in the 18 months post-MAP entry.

d. *P* tested using the Student's *t*-matched pairs test.

* *P*<0.05,

** *P*<0.01,

*** *P*<0.001.

### Outcome scores and severity assessment

Outcome scores and severity assessments are shown only for cohort 1, owing to the small number of patients in other subgroups who completed follow-up assessments. The mean MARS score at each time point is given in Fig. DS1 (see the online data supplement to this paper). In the 84 patients with MARS scores recorded at both baseline and 15–18 months, a 2.24 point improvement (s.d. = 2.14, *P*<0.001) was noted.

CGI severity was available for 93 patients at baseline and 83 patients at 15–18 months; online Fig. DS2 shows the category distributions. At 15–18 months, 47% (39/83) of patients had a less severe rating, 11% (9/83) a more severe rating and 42% (35/83) the same rating as at baseline, a significant shift towards less severe CGI categories (Wilcoxon, *P* = 0.004).

### Medications for psychiatric conditions

Prescription data were collected for 96% of patients. There were few medication changes during MAP participation: 6 patients switched from depot to oral antipsychotic medication and one switched from oral to depot.

### Patient and staff satisfaction

At least one satisfaction questionnaire was completed by 81 patients (80 in cohort 1, 1 in cohort 2). Patient satisfaction was high across all questions: 96% (75/78) of respondents ‘strongly agreed’ or ‘agreed’ that they would recommend MAP to a friend with a similar problem (see online Fig. DS3 for full results).

Eight staff who were involved in the delivery of MAP were interviewed: 4 mental health nurses, 3 occupational therapists and 1 consultant psychiatrist; 3 had worked on MAP since its initiation (>12 months), 4 for 6–12 months and 1 for <6 months. Two interviewees had left the service at the time of the interview. Themes identified in the qualitative analysis of interview responses are summarised below.

#### Positive aspects of the programme

MAP enables staff to spend more time with patients and develop a rapport, thus removing professional boundaries and resulting in a more equal, collaborative relationship. This is reinforced by the emphasis on customer service, care and respect, and the pleasant surroundings of the premises. Staff are encouraged to provide a truly client-centred service to enable shared decision-making. In-depth psychoeducation gives patients valuable knowledge and skills to become independent and empowered.

MAP has positive effects on job satisfaction, sickness absence and personal well-being. It gives staff more time and freedom to respond to patients' needs, enabling them to ‘do the job they were trained to do’ and make a real difference. Working on the programme is challenging and educational, with opportunities to acquire new skills, increase knowledge and share ideas through multidisciplinary team-working.

‘MAP magic’, a phrase coined by one staff member to describe the programme's impact, was described as a ‘positive change’ occurring in both patients and staff; patients become more empowered and less dependent and consequently, staff become happier and more fulfilled. This was described as a ‘cycle of positivity’, ‘positive feedback loop’ and a ‘connection’ between patients and staff occurring as a result of their changing relationship.

#### Negative aspects of the programme

The lack of administrative support for delivering MAP and difficulties in identifying and recruiting patients were the only negative aspects raised by more than one participant.

## Discussion

The gender distribution in the evaluation sample (55% male, 45% female) is consistent with previously published work which suggests that schizophrenia and bipolar disorder affect men and women equally.^13,14^ Data were not available on the duration of diagnoses prior to MAP, but the mean age at MAP entry (45 years) and most common age at disease onset (16–30 years in schizophrenia and before age 25 in bipolar affective disorder)^13,15^ suggest that for most patients diagnosis pre-dated MAP entry by many years. Bipolar affective disorder appears to be underrepresented in the sample (27% of patients *v.* 72% with schizophrenia or schizoaffective disorder), given that anecdotally, approximately equal proportions of patients with these conditions are treated within SEPT. This discrepancy may be explained by an existing and already well-established out-patient model of care within the trust for patients with bipolar affective disorder or the generally lower resource use in patients with this condition.^6^ There were some apparent differences between the two evaluation cohorts; a slightly higher proportion of patients in cohort 2 (14% *v.* 8%) were referred from an in-patient ward and had Mental Health Act applications prior to MAP entry (40% *v.* 24%). This indicates a more difficult to engage group and suggests that there may be benefit in reviewing MAP screening criteria, particularly the appropriateness of trying to engage patients from an in-patient ward. More patients in cohort 2 were male (67% *v.* 48%), also in line with previous findings that male patients are more difficult to engage and at higher risk of non-adherence.^16^

### Programme duration and dropout rate

Given the nature and severity of their illness, it would be expected that a proportion of patients would drop out of MAP. The 30% who left within 18 months is in line with the dropout rates reported in other studies of schizophrenia, which vary from around 13% for psychosocial interventions to 43% for placebo-controlled studies.^17,18^ However, although the evaluation period was 18 months, the duration of a ‘completed’ MAP has not been defined and even in cohort 2, some patients attended a number of sessions (mean 4.14 contacts, range 1–22). Although they may have benefited, it is important for future service provision to understand why these patients left, especially since pre-MAP resource use was highest in this group.

### Resource use

In-patient resource use reduced significantly post-MAP entry (mean reduction 24 bed days per patient overall; 26 days in cohort 1) without concomitant increases in other emergency resource use. In fact, in the cohort that remained in MAP for 18 months (cohort 1), assessment unit attendances also decreased significantly and there were non-significant reductions in CRHT, assertive outreach and CMHT duty team usage. There are several benefits of MAP and specifically psychoeducation which may have contributed to this: the improved therapeutic alliance, better self-management of symptoms, improved medication adherence and functional outcomes which in turn result in reduced symptom severity and lower risk of relapse and hospitalisation.^19^ Irrespective of the cost impact, this shift from ‘crisis’ to planned and potentially community-based care is valuable both for patients and the trust. The evaluation also shows that patients continue to engage with CMHT during MAP, which is important, since MAP was intended to supplement usual care.

The net cost reductions of £8253 and £7688 (overall sample and cohort 1, respectively) are largely in line with the evaluation of the Munich Adherence Programme, which showed estimated savings of €5000 per patient per year,^10^ although direct comparison is difficult due to differences in healthcare systems, observation periods and resource parameters. Furthermore, in the Munich pilot, unlike this evaluation, approximately 40–50% of patients were switched from oral to depot antipsychotics and it is not known how much this contributed to the results.

### Outcome scores and severity assessment

In cohort 1, MARS scores increased significantly post-MAP and importantly, moved out of the range indicating poor medication adherence (<6). There was also a shift towards less severe CGI categories, suggesting improved outcomes for patients. The largest increase in MARS scores occurred in the first 3–6 months of the programme and these early improvements were sustained throughout the evaluation period. The benefit to patients of the smaller score changes occurring later in the programme (between 6 and 18 months) are unclear and further qualitative research would be required to investigate this. Collection of scores after discharge from MAP is also needed to demonstrate whether the improvements are maintained.

### Patient and staff satisfaction

Patient and staff satisfaction was a key component of the evaluation and is vital in demonstrating MAP's long-term sustainability. Patient satisfaction was very high, with 96% saying that they would recommend the service to friends and family. Direct comparison of patient satisfaction with this programme and other mental health services is problematic owing to differences in questionnaires and respondent demographics, however, in a recent national survey of people using NHS community mental health services in England, 80% of respondents rated their overall care as excellent, very good or good.^20^ Although patient satisfaction in our evaluation appears to be higher than national figures, questionnaires were only available for those who continued in the programme and were likely to be the most satisfied. Qualitative interviews with patients who left MAP would provide valuable insight into their views on the service, the reasons for leaving and why it failed to continue to engage them.

Staff were overwhelmingly positive about the programme, describing many benefits from it for both patients and staff. Negative aspects mainly related to a lack of administrative support for delivering MAP rather than the programme itself. The importance of staff satisfaction was highlighted in a recent review which demonstrated better trust outcomes (patient satisfaction, mortality, infection rates, annual health check scores, staff absenteeism and turnover) when staff are engaged.^21^ The authors of the review concluded that a culture of engagement, positivity, caring, compassion and respect provides the ideal environment for patient care, and these emerged as strong themes in our staff interviews. In considering the link between staff and patient satisfaction, the authors cite the ‘symbiotic’ relationship of staff and patient experience whereby staff who are aware that patients are satisfied are more likely to view quality of care more positively themselves. The cyclical nature of the patient–staff relationship was also a key theme in the current evaluation.

### Limitations

This was a local evaluation, not a formal research study. The results are intended for local use and cannot be generalised. The sample is relatively small and was chosen to provide a sufficiently reliable estimate of the in-patient bed reduction in the overall sample, not to detect differences in other resource parameters or between cohorts.

There was no formal control group as there are ethical and practical issues in providing a control arm within the trust. However, patients acted as their own controls. It could be argued that as patients with one or more in-patient admission (or similar) were selected for the programme, a reduction in resource use would be expected irrespective of MAP. However, this is unlikely in these chronic and severely ill patients. Furthermore, an 18-month evaluation period was chosen to ensure that natural fluctuations in resource use would be observed in both periods.

The benefits of the programme may be overestimated since prospectively completed clinical measures and questionnaires are only available for those who remained in the programme, rather than the whole ‘intention-to-treat’ population. Also, MARS measures ‘propensity to adhere’ rather than actual adherence, which is notoriously difficult to measure in any setting.

Overall resource use and costs may be underestimated since no medication costs have been included. Furthermore, the evaluation includes only resources consumed within SEPT and we realise that patients may have visited other hospitals. However, these apply equally both pre- and post-MAP entry.

Medication data were collected retrospectively from patients' medical records; this relies on the completeness of these records.

The evaluation was designed to capture the impact of MAP when delivered as a package and does not show whether improved outcomes resulted from specific programme modules, improved therapeutic alliance or the optimal combination/duration of modules. A randomised study would be needed to assess the relative impact of each component.

### Final word

Central to MAP is evidence-based psychoeducation and targeted support activities within a supportive and positive environment which aims to help patients stay out of hospital and live better with their condition. This evaluation shows that in this real-world environment, introduction of this multifaceted and evidence-based programme to encourage adherence in patients with severe mental health disorders was associated with reduced cost of treatment, improvements in clinical outcomes and very high patient and staff satisfaction.
